# Dr. Drake, on Digitalis

**Published:** 1800-12

**Authors:** Nathan Drake

**Affiliations:** Hadleigh, Suffolk


### *To the Editors of the Medical and Phyfical Journal.*

GENTLEMEN,

I Enclofe you a further ftatement of the refult of my practice with the Fox-glove in Pulmonary Confumption. It has, hitherto, anfwered all my expectations; and I am every day more and more convinced of the decifive falutary powers which this medicine exerts over a difeafe which has, until very lately, baffled all the efforts of our art.

Confcious of the rectitude of my intentions, and of the accuracy with which my details have been given to the public, I fhall prefs forward, totally regardlefs of what *petulance* and *abuse* may fcatter in my path.

To the objections of any individual, when propofed with liberality and in the fpirit of improvement, I am ever ready to liften liften with deference and attention; but *mere fcurrility* neither merits nor admits of a reply. I am,

GENTLEMEN,

With great refpect,

Your moft obedient fervant,

*Hadleigh, Suffolk* NATHAN DRAKE.

*November* 6, 1800.

---

My views in publifhing the two cafes of Marris and of Grimes, were to aroufe the attention of the medical world to the fingular and hitherto unobferved powers of the Fox-glove in confumption; by this mean, a body of evidence, either for or againft the utility of the medicine, might fhortly be expected. The Digitalis had previoufly been exhibited in this complaint but in very few inftances; thofe recorded by Dr. Darwin being the only cafes, I believe, known to the public; and in thefe, the plant was exhibited merely with the view of promoting temporary ficknefs and confequent abforption.

My intentions in prefcribing the Fox-glove in phthifis were, however, widely different; and the refult of the trial evinced an energy equally novel and unexpected. It was my wifh to produce its full influence on the circulating fyftem, and this too, gradually and independent either of naufea or ficknefs, yet productive of rapid abforption: Thefe objects I obtained; and in effecting them, difcovered, that by a cautious ufe of the faturated tincture, the circulation might not only be depreffed, and that to a very confiderable degree, but for any given length of time, and without much inconvenience, or any danger to the patient. Two gentlemen labouring under confirmed phthifis were, under this plan, completely cured; and I thought it my duty to announce to the world, as foon as poffible, events fo unexpectedly fortunate. I alfo ventured to draw from the refult of thefe two cafes, the following inference, that as the Digitalis had re-eftablifhed health in the moft confirmed ftate of phthifis, it might, in its earlieft ftage, be expected *generally* to prove fuccefsful.*

That I have acted thus; that I have publifhed thefe cafes, and drawn this inference, is to me, in the prefent ftage of my experience with the Fox-glove, a matter of the higheft felf congratulation. To ftate this experience, forms the principal object of the prefent paper; but, before I enter upon it, it will be neceffary to premife a few obfervations.

I have been for near ten years in the habit of prefcribing the powder

---

* Vide Contributions to Phyfical and Medical Knowledge.

powder of Digitalis in anafarcous cafes; my friend and predeceffor, Dr. Gibbons, for better than fifteen: Our experience has taught us, that, although it will fometimes ftrongly influence the circulation, independent of any action on the ftomach, yet it is very apt, even in the moft guarded dofes, to produce exceffive naufea and ficknefs, effects very frequently ufeful, and even neceffary in promoting its diuretic action: As, however, to induce this action was no object in my view, and as I particularly wifhed to avoid its effects on the ftomach, and at the fame time to introduce it gradually, and for a long period into the fyftem, I chofe the faturated tincture as the preferable preparation; and as this has anfwered all my expectations, I have not been tempted, except in one inftance, which will be mentioned hereafter, to exchange it for any other.

Even the faturated tincture will too frequently affect the ftomach, if not given in the moft cautious manner; and I have obferved, without a fingle exception in *my* practice, that ficknefs has uniformly been pernicious to the patient, and has ever interrupted the progrefs of the cure. To obviate this, I have lately given the tincture in fo very gradual a manner, that the patient ufually takes it a fortnight or longer, before any decifive effects are felt on the fyftem. I increafe the dofe but by one drop per day, or at moft, by one drop per dofe; and I have now a patient under my care who is taking eighty-eight drops daily, and who, for thefe laft three months, has taken from fifty to this quantity per day, without even the fmalleft naufea, and without any affection of the head, which could be termed troublefome, although his pulfe, which, before the ufe of the tincture, beat about 100 ftrokes in the minute, has been during this period never, upon the average, above 60; once as low as 36, frequently not more than 50.

I continue to ufe the faturated tincture made in the proportion of five ounces of proof fpirit to one ounce of the coarfely powdered leaves. A few ounces of what was made laft year are ftill in my poffeffion; not the leaft depofition has taken place, and the colour is as deep, and the tranfparency as great, as when the tincture was firft filtered.

This year I have gathered, along with Meffrs. Travis and Bunn, a large quantity of the plant; and as we had an immenfe variety to felect from, we chofe only the fineft fpecimens, and, I believe, brought home none under the height of five feet; a great number was, I am convinced, more than fix feet high, fome much higher; the talleft plant, and in every refpect the fineft, from the number of its lateral branches and the multiplicity of its leaves, meafured, when frefh gathered and independent of its root, feven feet ten inches. The moft vigorous vigorous plants we have obferved invariably to grow where the deepeft fhade is afforded them; and this might be expected indeed, exclufive of individual authority, from the fituation the Digitalis holds in the natural order of the Linnæan fyftem, the Luridæ. Under this arrangement it has for *Congenera* the *Solanum Lethale*, the *Hyofcyamus*, *Datura*, &c. plants well known to thrive only beneath the protection of much fhelter.

In the 8th Number of this Journal I have mentioned, that no opportunity of exhibiting the Digitalis in incipient tubercular confumption had, at that time, occurred to me. Since that period, however, I have attended four patients previous to the commencement of the fuppurative procefs, and fix in whom a copious purulent expectoration had for fome time been eftablifhed. I now proceed to ftate thefe cafes and their refult, to which I fhall occafionally fubjoin a few obfervations.

Case I. *Martha Howlett*, of Dedham, Effex, aged 28, has been fubject to a violent cough for two years, and has now, March 29, 1800, when fhe applied to me, a pulfe beating 120 in the minute. Her expectoration amounts to about a tea-cup full in twenty-four hours, but has no purulent appearance; her breathing is very difficult, and fhe has had for fome months profufe colliquative perfpiration; her cough is almoft perpetual, and her emaciation is very great: fhe complains of cold fhivering fits, and of being frequently flufhed after her meals; appetite gone; great languor; thirft confiderable, but the tongue not foul; body regular; menfes regular; urine of a natural colour; fleep much difturbed.

I ordered her ten drops of the tincture of Digitalis twice a day; and in a letter to her furgeon, Mr. Fox, of Dedham, requefted they might be gradually increafed. On the 31ft of May, 1800, I received a letter from Mr. Fox, from which I have taken the liberty of extracting the following paffage:

"I have purpofely delayed giving you an account of Martha Howlett, until I could offer you my congratulations on the fuccefs of the Digitalis you prefcribed for her.

"The pulfe now continues fteadily under 60; her cough has nearly ceafed; her breathing is free and eafy; and her appetite and ftrength appear reftored to their healthy ftandard; a tendency to fyncope, however, and fome depreffion of animal power, rendered neceffary a retrogade dofe in the exhibition of the Digitalis. I have not yet been able to adminifter more than fifty drops in the twenty-four hours. I now give her only thirty every night; but which I mean again to advance, until the cough has totally ceafed.

"But, to do you proper juftice, I have fent her, that you may judge for yourfelf. Indeed, I have no doubt whatever that that the Digitalis has thus far removed a malady, whofe formidable degree gave me every reafon, from obfervation of paft events, to believe that death would, as ufual, foon inevitably have finifhed the fcene."

I agreed with Mr. Fox in the propriety of again increafing the dofe of Digitalis; and in a fubfequent letter he informs me, that "he could never exceed thirty-five drops twice a day, and that but for a very fhort time; thirty, twice a day, was the ufual dofe, which fhe continued TEN weeks; her pulfe was never depreffed much below 60."

Towards the latter end of Auguft I called upon her, and was happy to find her reftored to full vigour and health, free from every complaint except an hoarfenefs, which fhe had acquired a week or two before I faw her, by expofure to cold in wafhing.

CASE II. *Mifs Bridgman*, aged 27, of ———, near Difs, in Norfolk, has for feveral years been fubject to a very great, conftant, and almoft fingular rapidity of circulation, her pulfe ufually beating from 120 to 130 ftrokes in the minute; fhe has likewife had for feveral years a moft troublefome and very frequent cough, attended with great difficulty of breathing upon motion. There has never been any expectoration, but the proftration of ftrength and the emaciation are extreme; the nightly perfpirations alfo are frequent, but not very copious; fhe has little or no appetite, and has lately been fubject to diarrhœa, which is now only checked by the ufe of opiates. Menfes deficient. She has had the advice of many eminent practitioners.

Shortly after her arrival at Hadleigh fhe was placed under my care, and I ordered her, on April 19, 1800, ten drops of the tincture of Digitalis twice a day, in a flight infufion of quaffia and cinnamon, and an opiate pill at bed-time. The tincture was cautioufly increafed for two-and-twenty days, until the quantity taken in twenty-four hours amounted to fifty-two drops, and the pulfe gradually, during this period, fell to 80.

Under the exhibition of the Digitalis, languor and occafional naufea were felt; and, at length, when the pulfe had dropped to 80, fo much tendency to fyncope took place immediately after each dofe, that I thought it neceffary to difcontinue the tincture. The cough, however, had been for fome time much better; the irritation was greatly mitigated; the appetite increafed, and the ftrength evidently improved; nor did her pulfe, upon the omiffion of the Fox-glove, acquire its former velocity, though it rofe much beyond the healthy ftandard.

I now prefcribed for her a preparation of cinchona, myrrh, and prepared kali, in the form of a draught, twice or thrice a day; day; and after each dofe fhe was directed to take a table fpoonful of lemon-juice. This agreed very well; her appetite became keen, and the powers of mufcular motion increafed. In this fituation fhe returned home, and I requefted her furgeon, Mr. Harrifon, of Difs in Norfolk, to refume the Digitalis as foon as her ftrength fhould be fo far improved as to admit of its being fafely tried. This gentleman wrote to me occafionally; and his laft letter, dated October 7, includes the following fatisfactory account:—

"I am particularly happy in communicating to you Mifs Bridgman's amendment, which has taken place fince her commencement with the Digitalis, (TEN weeks fince): for the laft three weeks fhe has taken thirty drops twice a day; no unpleafant fymptoms have occurred from it; I have not found the pulfe more than eighty for this laft month, twice in that period not quite fo many; from increafe of bodily ftrength, fhe is able to take more exercife, and without fatigue; her cough is lefs troublefome, her breathing lefs laborious, and her appetite good, with every fymptom of increafed health.

"November the 5th. I have this day heard from Mifs Bridgman, through the medium of her brother; fhe continues the Digitalis, which fhe has now taken FOURTEEN weeks; her health has progreffively improved fince October the 7th."

The very rapid, conftant, and long continued quicknefs of circulation is, in this cafe, moft remarkable; I could not have previoufly conceived, that a pulfe of near 120 could, for fo extended a period, have been compatible with life; nor did I think the Digitalis would have exerted an influence fo decided and falutary, where the circulating fyftem had been fo long habituated to movements of fuch unufual velocity.

CASE III. *Sarah Hammond*, aged 17, complains, June 13, 1800, of great difficulty of breathing, great ftricture acrofs the cheft, and of pain in her left fide; pulfe near 120, fkin hot, thirft not confiderable; has cold fhivering fits twice a day, and copious colliquative perfpirations toward morning; has little appetite; her ftrength is much impaired, and the emaciation confiderable; not much cough, and no expectoration; tongue foul, and of a brown colour; body very coftive. The catamenia have not appeared for three months. She has been twice attacked with fimilar fymptoms, which were removed, under the care of Mr. Travis of Bergholt, by tonics and preparations of fteel. She has loft a fifter in confumption, whom I attended fome years ago.

Mr. Travis, when called in on June 13, ordered her, in the firft place, a purging draught, then a faline mixture with myrrh, myrrh, and afterwards ſome tonic pills with ſteel; ſhe had alſo a bliſter on her ſide, and was bled; the blood appeared ſizy.

As no benefit was derived from this plan, he ordered her, on June 30, ſeven drops of the ſaturated tincture of Digitalis thrice a day; theſe were gradually increaſed to ſixteen thrice a day, and ſhe was under the influence of the medicine for a month. During this period her ſymptoms gradually diſappeared, and her pulſe ſunk to the ſtandard of health. She experienced no ſickneſs, nauſea, or giddineſs of the head, but complained of being frequently very ſleepy.

For the particulars of this Caſe I am chiefly indebted to Mr. Travis, as I had an opportunity of ſeeing the patient but once. On September 2, ſhe was, in all reſpects, perfectly well.

CASE IV. *Mrs. Salmon*, of St. Oſyth, came to Hadleigh on October 2, 1800, to conſult Dr. Gibbons and myſelf. She has been ſubject for ſeveral months to a violent and frequent cough, with conſiderable difficulty of breathing and profuſe colliquative perſpirations; has frequent ſhivering fits. There are much emaciation and great debility preſent. Pulſe 124, and weak; ſkin hot; body regular; urine natural; appetite nearly gone; no expectoration.

We ordered her ten drops of the tincture of Digitalis twice a day, in an infuſion of quaſſia and orange-peel, to be gradually increaſed to twenty, morning and evening.

October 21ſt. Though only nineteen days have elapſed, nearly all her ſymptoms have diſappeared, and her countenance has the appearance of health. We have ſeldom witneſſed a more rapid amendment. Her pulſe beats but 66 in the minute, and her breathing is perfectly eaſy. The cough is gone; the perſpirations are removed; the appetite is keen; the ſtrength greatly improved, and ſhe takes ample exerciſe daily without fatigue. Has felt ſome giddineſs and tremor after taking each doſe of the medicine, but no nauſea.

Of theſe four Caſes of incipient phthiſis, the firſt, the ſecond, and the fourth, I conſider as examples of phthiſis ſcrophuloſa; the third, an inſtance of phthiſis chlorotica. With regard to the ſecond caſe, though I am convinced, from every ſymptom, that tubercles had long exiſted in the lungs, yet I am of opinion, had the Digitalis not been interpoſed, and the diſeaſe had been ſuffered to run its courſe, that the purulent ſtage would, nevertheleſs, not have ſupervened, but that the patient would have been gradually worn down by the continued rapidity of the circulation, and the increaſing cough and difficulty of breathing.

I now proceed to thofe cafes in which the purulent ftage had been for fome time eftablifhed.

Case V. *Anne Bush*, of Dedham, aged 28. May 14th, 1800; has been ill for fix months, and during that period, gradually getting worfe; fhe complains now of great difficulty of breathing, of violent cough, and of acute darting pain in her left fide; her pulfe is weak, and beats 110 ftrokes in the minute; fhe has had for feveral weeks a heavy purulent expectoration, amounting to about half a pint in twenty-four hours; has frequent fhivering fits, and copious colliquative perfpirations: the leaft exertion exhaufts her, and fhe is reduced to a mere fhadow; thirft confiderable; appetite nearly gone; fkin dry and hot; and fhe is much flufhed in an evening; her menfes have been for fome time fuppreffed.

I ordered her eight drops of the faturated tincture twice a day, and thefe were foon increafed to twenty per day; but the morning dofe then occafioning fome giddinefs, I requefted the twenty drops might be given only at night. She never reached more than thirty drops per night, but the effect upon the circulating fyftem was great and permanent. On the 31ft of May, her furgeon, Mr. Fox, informs me by letter: "Your patient, Anne Bufh, I confider much better; her circulation is much flower, and the cough lefs. I have not been able to give her more than twenty drops in twenty-four hours, which I now give at night, as you directed." Soon after this period fhe increafed her drops to thirty; but on the 7th of July, Mr. F. tells me, "I have lowered the dofe of Digitalis to twenty-five drops every night, as your patient has complained of much giddinefs. Her cough and expectoration are now but little, and her circulation fteadily at or under 60; but the pain in her fide is ftill troublefome, notwithftanding repeated blifters; I therefore recommended her to fee you once more."

I directed an iffue to be cut in her fide, the difcharge from which, in a fhort time, relieved her pain. She was under the influence of the Digitalis for SEVEN weeks; and fhe took it, latterly, in decoction of bark, making ufe at the fame time of a generous diet. She is now, Auguft 30th, 1800, perfectly well, with a pulfe of 68. I faw her during this period fix times. She experienced, though the quantity of tincture was ever fmall, a confiderable degree of languor and much giddinefs, and had ufually an intermitting pulfe.

Case VI. June, 1800. *Anne Hart*, aged 16, has a copious purulent expectoration, amounting to better than half a pint per day; pulfe 128, fmall and weak; night fweats profufe; cough violent, with great palpitation of the heart and difficulty of of breathing; complains of ſhivering fits morning and evening; ſkin hot, body regular, thirſt conſiderable, appetite totally deſtroyed, emaciation extreme, and debility ſo great as to be generally confined to her bed. Has never menſtruated.

Her ſymptoms aſſumed a very ſerious aſpect in December 1799, at which period ſhe was ordered, by Mr. Travis of Bergholt, a mixture with myrrh and ſteel, which ſhe continued until February 27, 1800, with ſome benefit; but on April 5th, her complaints having returned with increaſed violence, he gave her the tincture of Digitalis in very ſmall doſes, which in a ſhort time mitigated, and for a few weeks nearly ſuſpended, the moſt preſſing and dangerous ſymptoms; ſhe relapſed, however, toward the latter end of May; and in June I ſaw her labouring under the circumſtances above deſcribed.

I requeſted ſhe might reſume the Digitalis, and continue it ſteadily for ſeveral weeks. She began with five drops three times a day, which were very cautiouſly increaſed to eleven three times a day, the greateſt number ſhe could ever take without ſickneſs.

July, 1800. She has taken the tincture for near five weeks, and appears to me greatly relieved by it, and her ſtrength is ſo much increaſed that ſhe has been able to walk to Mr. Travis's to meet me. Pulſe 100. Ordered to continue the tincture.

September, 1800. Met her again at Mr. Travis's; ſhe has regularly taken the tincture ſince July; her pulſe is now only 90, and has never, I underſtand, dropped lower; cough entirely removed; expectoration has for ſeveral weeks diſappeared; has had no perſpirations for a month; appetite good; ſleeps well; body regular; ſkin cool; ſtrength improving; but has gained little fleſh.

November 3d, 1800. She is now ſo much altered for the better in her perſonal appearance, that it was with ſome difficulty I recollected her. She is free from every complaint, and her ſtrength and fleſh are perfectly reſtored. Pulſe 64. She has not taken any Digitalis for this month paſt.

Case VII. *Mr. Hollick*, of Stratford, aged 22, labours under a frequent and very troubleſome cough, attended with an expectoration evidently purulent, and amounting to about a tea-cup full in twenty-four hours. Pulſe near 100; great emaciation; much debility; colliquative perſpirations towards morning; complains of pain and uneaſineſs in the cheſt, but not of much difficulty of breathing, except upon motion; little thirſt; tongue clean; appetite much impaired; ſkin hot; body regular; urine natural; ſleep diſturbed; frequent ſhivering fits: Theſe ſymptoms acquired their preſent alarming growth in about three months.

My

My advice was requested on July 25, 1800, when I ordered him ten drops of the saturated tincture of Digitalis night and morning; at first in an infusion of quassia, afterwards in an ounce and a half of the decoction of cinchona with two drachms of the tincture of orange-peel, and occasionally with the addition of a few drops of the nitric acid, and a slight opiate at bed time. The following dates denote the days on which I visited him, between which his surgeon saw him daily, and paid him the most marked attention; to this gentleman, indeed, I am particularly indebted for the skillful manner in which, during my absence, he increased or diminished the quantity of the Digitalis, according to circumstances.

August 2, 1800. Dose of Digitalis increased to forty drops per day. Pulse 48, soft and steady; skin cooler; cough not so troublesome; in other respects as before. He has no affection of the stomach or head. Ordered to live generously, to take a few glasses of Port wine after dinner, and to take gentle exercise every day on horseback.

August 9th. Digitalis increased to fifty-four drops daily; the pulse has risen, beating 60, soft, and with occasional intermission; no nausea or giddiness; expectoration rather decreased; cough much less; skin perfectly cool; perspirations trifling; appetite improved.

August 16th. Digitalis increased to sixty drops; pulse 68, and slightly intermitting; expectoration diminished; very little cough; appetite good; sleep undisturbed; perspirations removed; takes six glasses of Port wine and a bottle of cyder daily, and rides out every afternoon two miles on horseback.

23d. Dose of Digitalis per day sixty drops; pulse 70; expectoration increased again to a tea-cup full, and very purulent; appetite continues good; no nausea or giddiness; takes ten glasses of wine daily; no perspirations, and not much cough. Ordered the tincture of Digitalis to be increased.

30th. Dose of Digitalis seventy-two drops; pulse 60, and intermitting; expectoration again diminishing; strength not decreased; takes a bottle of Port wine per day, and rides out as usual.

September 6th. Dose of Digitalis seventy-six drops; pulse 58, intermitting; expectoration still continues purulent, and with regard to quantity stationary; appetite good; skin perfectly cool, though taking better than a bottle of wine daily; complains of pain in his side, which appears to be merely muscular. Ordered a volatile liniment to be applied to the pained part.

13th. Dose of Digitalis 80 drops; pulse 54; expectoration much diminished; cough nearly gone; pain of the side removed; moved; feels himfelf ftronger; breathing eafy; fkin cool; continues his wine; appetite as ufual.

20th. Dofe of Digitalis as before; pulfe 56; expectoration nearly removed, and no longer purulent; cough gone; takes a bottle and a half of Port wine per day; fkin cool; complains of finking and languor, which are relieved by taking wine frequently; requefted to do fo whenever thefe fenfations recur; ftrength improving, and rides out much further without fatigue; has five drops of tinctura opii in each draught.

October 9th. Since September 20th, his pulfe has rifen to more than 70; and his expectoration returning, the tincture was again increafed, the dofe being now 88 drops; pulfe 48, and intermitting; expectoration about a table fpoonful; has gained fince my laft vifit fome flefh, and his ftrength is greater; but now complains of a fenfation of twifting in his bowels after each dofe of his medicine, and of much finking at the pit of his ftomach; takes two bottles of Port wine per day; fkin cool; his head not in the leaft affected by the wine; appetite good; body regular; no perfpirations; no pain; no cough; no naufea; no giddinefs; rides out as ufual.

October 23d. Dofe of Digitalis eighty-eight drops per day; pulfe 58 and regular when laid upon his couch, but much accelerated upon motion; no cough; breathing perfectly eafy; expectoration not a table fpoonful, and little if at all purulent; uneafy fenfation in his bowels removed; no perfpirations; appetite not quite fo good as on October 9th; took yefterday near three bottles of Port wine; ftrength and appearance as at the laft vifit; body regular; fkin cool; fpirits very good; no naufea; no giddinefs. Ordered to continue the Digitalis, and to take a little nitric acid in each draught.

November 3d. Since Oct. 23 his pulfe has funk to 36, and the intermiffion being alarming, the Digitalis was omitted for two days, and then refumed at the rate of twenty drops morning and evening; the dofe is now fixty drops per day, viz. thirty morning and evening; pulfe 64, foft and regular when laid upon his couch, but intermitting, though not accelerated, upon motion. On the reduction of the Digitalis no neceffity was found for the quantity of wine he had been accuftomed to; he now takes only one bottle per day. He experienced no ficknefs even under the great depreffion above mentioned. Expectoration about a table fpoonful; ftrength and appearance as on Oct. 23; fkin cool; fpirits good. Ordered to continue the Digitalis.

This very inftructive cafe, whofe termination I muft now leave open, but which fhall be communicated in a future Number, proves what a confiderable degree of ftimulus may be borne borne with fafety and utility, when the fyftem is under the full influence of the Digitalis. Not only whilft taking two bottles of wine per day was the pulfe unaltered, and beating but between 50 and 60 ftrokes in the minute, but the fkin alfo remained perfectly cool, nor was he at any time flufhed, nor was his head in the leaft affected.

Before he commenced the ufe of the Fox-glove a fingle glafs of wine flufhed him, and his appetite was nearly gone; his cough was violent; his expectoration confiderable; his night fweats profufe; his pulfe quick; the uneafinefs in his cheft much, and his fleep greatly difturbed.

He is now, November, 1800, free from fever; his pulfe being now, and having been for near three months, upon the average, but 60 in the minute; his appetite is good, and two bottles of wine per day neither heat him nor affect his head; his cough is gone; his purulent expectoration a mere trifle; his breathing eafy; his perfpirations completely removed; his fleep found; and he has neither pain, ficknefs, nor giddinefs; he rides out every day; and his fpirits are, and have been, whilft taking the medicine, perfectly good and equal.

Notwithftanding, however, this favourable change, fome fymptoms, indicative of danger, remain; his expectoration, though greatly diminifhed, ftill continues; he gains little flefh, and there is much debility; and frequently much acceleration of his pulfe on motion. In this ftate of the difeafe, I do not choofe to commit myfelf by prefuming to predict the event.

CASE VIII. *Mr. Smith*, of Semer, aged 50. September, 1799, has a very copious purulent expectoration, a violent cough, and profufe colliquative perfpirations; ftrength fo much diminifhed as to be confined to his bed; pulfe 100. He has been ill better than two months, and has loft two fifters and a brother by the complaint: he has taken the tincture of Digitalis, under the care of his furgeon, Mr. Growfe, of Bildefton, and reached ninety drops per day, forty-five morning and evening, when his pulfe had funk from 100 to 50 ftrokes in the minute, without any confequent giddinefs, and with very little naufea. He continued the Digitalis for three weeks, during the latter part of which, his expectoration, Mr. Growfe informs me, was much decreafed, and his cough greatly better; his perfpirations were lefs copious, and he acquired fo much ftrength, as to get down ftairs and take fome exercife: at this period he left off the medicine, complaining greatly of the languor it produced. In four weeks after, however, his former fymptoms returned; and when I firft faw him, he had refumed the tincture by Mr. Growfe's direction, which I ordered to be increafed increafed gradually, until the pulfe fhould again drop to 50 in the minute, and to be kept at that ftandard for fome weeks; it was taken in an infufion of quaffia, along with fome tincture of myrrh.

By the commencement of October, 1799, the pulfe was again reduced to 50, accompanied by much languor and fenfe of faintnefs, but with no vomiting or giddinefs; the appetite was improved, the circulation had no intermiffion, the expectoration too was much diminifhed, and the cough lefs frequent.

In this ftate he refufed any longer to continue the medicine, notwithftanding our repeated and earneft folicitations, affirming he would rather die than endure again the languor he had experienced: I vifited him thrice, folely with the view of inducing him to perfevere, but in vain. What would have been the refult, had he fubmitted to our wifhes, I cannot pretend to fay; it is probable it might have been unfavourable; but, as independent of the Digitalis, he had no chance of recovery, and though exciting unpleafant feelings, it had been productive of no dangerous fymptoms, but had already, on the contrary, exerted a decided falutary power; I thought it my duty to urge a longer trial. He lived, I believe, three or four months after relinquifhing the Fox-glove.

I have been the more particular in this cafe, as it affords a ftrong example of what obftacles fometimes oppofe the exhibition of this medicine. There is no arguing againft fenfation; and it were much to be wifhed, that either fome corrective were difcovered, or fome fubftitute of equal power over the heart and arteries, yet ftript of the tendency to affect the head and ftomach. I have reafon to believe, however, from the cafe of Mr. Hollick, that by exhibiting the tincture of Digitalis in the moft gradual manner, fo as to habituate the fyftem to its action, thefe troublefome confequences may be altogether fuperfeded, and the circulation, notwithftanding, brought fully under its influence. The cafes too of Marris, Grimes, and Ames, are in confirmation of this idea.

Case IX. *Mrs. Green*, of Hadleigh, aged 20, was attacked many months ago with violent cough, great difficulty of breathing, and flight mucous expectoration; fhe had alfo colliquative perfpirations, and morning and evening exacerbations. Thefe fymptoms were checked, and for a time altogether fufpended, by the progrefs of pregnancy; but in a week after fhe was brought to bed they re-appeared with double violence, and the expectoration became loaded with pus. Two of her fifters died of pulmonary confumption.

I ſaw her on September 25, 1799, when ſhe had been taking for ſome time fifty drops per day of the ſaturated tincture of Digitalis, under the direction of Mr. Bunn, and her pulſe, which had been previous to the uſe of the Fox-glove 112, now beat but 80 ſtrokes in the minute; her cough, difficulty of breathing, and perſpirations are better; the expectoration, however, ſtill continues copious, and very purulent.

I requeſted the Digitalis might be gradually increaſed, and taken thrice a day; this was done, firſt in a preparation of cinchona and vitriolic acid, afterwards, when ſickneſs took place, in lemon juice, which enabled her to retain the drops on her ſtomach. The largeſt quantity ſhe could ever take of the tincture amounted to ſixty-ſix drops per day, divided into three doſes, at which time her pulſe dropped to 60, and for a ſhort period the greater part of her ſymptoms, even the expectoration, vaniſhed, and I entertained ſome hope of a ſalutary reſult. This amendment was, however, but tranſient, for, by the 16th of October, the ſymptoms returned with accelerated force, and ſhe died before the expiration of the month.

CASE X. *Mrs. Holton*, of Nayland. Of this caſe I have preſerved no memoranda, having only ſeen her once, and when ſhe was in the laſt ſtage of phthiſis. She had taken, however, the tincture of Digitalis under the eye of Mr. Harrold, a very intelligent practitioner of the place. Only tranſient benefit had been received from it; and when my advice was requeſted, on December 26, 1799, ſhe was in a ſtate of very great debility. I ordered her the powder of Digitalis in pills, with a ſmall quantity of opium purificatum, but little or no relief, I believe, was obtained. She died, if I recollect aright, about two months after I ſaw her.

CASE XI. *Mr. Green*, of Boxford, applied to me on the 9th of June, 1800. He is of a conſumpitve family, and has been for ſome time affected with every ſymptom of tubercular phthiſis. His expectoration I have had no opportunity of inſpecting. I requeſted Mr. Growſe, his ſurgeon, and near relation, would try the effects of the ſaturated tincture of Digitalis; and, on June 18th, when I laſt ſaw him, the medicine had agreed well, and I thought him better. I have taken no notes of this caſe, neither am I at preſent acquainted with the reſult.

Of theſe eleven caſes, four were inſtances of incipient phthiſis, and were cured; indeed I have much reaſon to think, not only from my own experience, but from that of others detailed tailed in this Journal, that ſimilar ſucceſs will attend five caſes out of ſeven where ulceration has not occurred, and where the medicine has been early and ſkilfully exhibited.

Of the remaining ſeven caſes, ſix were certainly in the purulent ſtage; and of theſe, two were cured, and a third, viz. Mr. Hollick's caſe, remains undecided.

For the ſake of perſpicuity, and with a view of throwing the whole upon the eye at once, I ſhall give, in a tabular form, an arrangement of all the caſes of pulmonary conſumption in which I have attempted a cure through the medium of the Digitalis, ſtating the name, the ſtage, and the reſult.

| Names. | Stage. | Result. |
| --- | --- | --- |
| Marris, Mr. | Purulent. | Recovered. |
| Grimes, Mr. | Purulent. | Recovered. |
| Ames, Mr.* | Purulent. | Recovered. |
| Howlett Martha, | Incipient. | Recovered. |
| Bridgman, Miſs | Incipient. | Recovered. |
| Hammond Sarah, | Incipient. | Recovered. |
| Buſh Anne. | Purulent. | Recovered. |
| Salmon, Mrs. | Incipient. | Recovered. |
| Hart Anne, | Purulent. | Recovered. |
| Hollick Mr. | Purulent. | Undetermined. |
| Whitmore, Mr. † | Purulent. | Died. |
| Boutell, Mr. | Purulent. | Died. |
| Smith, Mr. | Purulent. | Died. |
| Green Mrs. | Purulent. | Died. |
| Holton, Mrs. | Purulent. | Died. |
| Green, Mr. | | |

From this table it will be immediately perceived, that out of ſixteen caſes of pulmonary conſumption, ſuppoſing Mr. Green's to have terminated unfavourably, nine have recovered, and one very important caſe remains yet undecided.

In thus ſtating hitherto the whole of my practice with the Digitalis in conſumption, I have taken much pains that every circumſtance ſhould be very faithfully recorded; with the view therefore of procuring greater accuracy, theſe caſes have been read over to moſt of the medical gentlemen who attended with me,

* The name of the patient whoſe hiſtory is given as Caſe III. Numb. VIII. p. 268.

† The name of the patient whoſe hiſtory is given as Caſe II. Numb. VIII. p. 267.

me, that any neceffary particular which had efcaped my minutes might obtain introduction.

It may be neceffary, however, before I conclude this paper, and with the idea of obviating any captious query, to ftate, that I have been fent for to fix patients who were literally dying, with a view of exhibiting the Digitalis! To two of thefe, who were actually in articulo mortis, I refufed, though preffed, to give one drop of the tincture; they both died, I believe, within forty-eight hours after my vifit. The third, Mrs. Ifaacs, of Witham, took about fourteen drops previous to her death, and, as might be expected, without any perceptible effect; fhe died four days after I faw her. The fourth, Mrs. Hooker, of Boxted, took alfo a fmall quantity of the tincture, but her debility being too great to bear its action, it was immediately given up; fhe died about a week after it was relinquifhed, and ten or eleven days after I faw her. The fifth, Jofeph Brett, apparently in a dying ftate, began the tincture, but it was almoft immediately withdrawn from its affecting his head; he died about ten days after I faw him. The fixth, Mrs. Hird, near Manningtree, in the very laft ftage of the complaint, under which fhe had laboured for near two years, attempted to take a fmall quantity of the tincture, but her ftomach could not bear it; fhe died a few weeks after.

I fhall now offer a few obfervations clofely connected with the fubject of this paper.

The power of diftinguifhing accurately between pus and animal mucus, has long been, and ftill is, perhaps, a defideratum. The following paffage from Mr. Everard Home's Differtation on the Properties of Pus, prefents, however, a curious, and, apparently, a decifive mode of afcertaining this diftinction: "The property," obferves he, "which characterifes pus, and diftinguifhes it from moft other fubftances, is, its being compofed of globules, (vifible when viewed through the microfcope.) Mr. Hunter was, I believe, the firft who took notice of this property; and has thereby furnifhed us with a very accurate diftinction between pus and animal mucus. For the appearance of what is properly termed mucus, that is, animal fubftance diffolved from putrefaction, is flaky, and very different in its appearance from pus. It is alfo by this property diftinguifhed from all the chemical combinations of animal fubftance that I am acquainted with, every one of which appear in the microfcope to be made up of flakes."*

I have

* On the Properties of Pus, pages 36 and 37.

I have noticed, in Numb. X. p. 417, of this Journal, the vaſt utility of the Digitalis in catarrh, vomica, and hæmoptoe; in this laſt dangerous diſeaſe I have never ſeen it fail. The following caſe, the lateſt which has occurred to me, is ſo ſtrikingly illuſtrative of the ſalutary effects of the Fox-glove in active hæmorrhage, that I ſhall make no apology for introducing it.

Mr. Ennalls, aged 27, and of a ſtrong robuſt habit, having by violent muſcular exertion ruptured a blood veſſel in his lungs, diſcharged, by coughing, nearly one pint of blood in twenty-four hours; his pulſe 120, firm and hard. He has taken the ſaturated tincture of Digitalis, by the advice of his ſurgeon, Mr. Growſe, of Bildeſton, twice and thrice a day; and, when the doſe amounted to ninety drops in twenty-four hours, the pulſe was reduced to 50, and the hæmorrhage entirely ſubſided.

In conſequence, however, of incautious exertion too ſoon after his recovery, the hæmorrhage returned with more violence than at firſt, and on May 2, 1800, my attendance was requeſted. He had bled profuſely; the diſcharge, however, had been ſomewhat checked by the reſumption of the Digitalis; his pulſe notwithſtanding beat 124 ſtrokes in the minute; the heat was great, the breathing difficult, and ſo much irritability was preſent, that the hæmorrhage recurred upon any the leaſt effort either of mind or body.

I ordered the tincture of Digitalis to be increaſed in as rapid a manner as was conſiſtent with ſafety, and until the pulſe ſhould again drop to 50; I likewiſe preſcribed a drachm of the nitric acid in a pint of water daily, and a regimen of the cooleſt kind to be ſtrictly obſerved, along with perfect reſt.

The pulſe in a few days, I underſtand from Mr. Growſe, was reduced to 48, and on May 13, when I ſaw him again, the hæmorrhage had compleatly ſubſided; the heat was natural, the breathing eaſy, and nearly every morbid ſymptom removed. Pulſe between 50 and 60, for the doſe of Digitalis had been lately decreaſed by Mr. G. Ordered to continue the tincture, but in reduced doſes.

Some weeks after this he had another ſlight attack, which was ſpeedily removed by again having recourſe to the Digitalis. He is now, September 1800, in perfect health.

I have had occaſion to mention, in a previous page, that an auxiliary to, or ſubſtitute for, the Digitalis, poſſeſſing its active ſalutary powers, yet with a minor portion of deleterious energy, would be an acquiſition of the firſt importance. May not ſuch a medicine be found in the *Rhododendron Chryſanthemum* of Linnæus? This ſhrub grows on the ſummits of the high mountains named Sajanes, in the vicinity of the river Jeniſca, in in Siberia, where, in a weak infufion, it is ufed for tea. A parcel of it was fent, fome years ago, to Edinburgh, and warmly recommended as a cure for rheumatifm. Dr. Home, in his "Clynical Experiments, Hiftories and Diffections," has given us a report relative to its effects in this complaint; it failed in alleviating the rheumatic fymptoms, but in every inftance exhibited a remarkable fedative effect on the heart and arteries. In three cafes recorded at length, it reduced the pulfe from 112 to 82, from 106 to 44, and from 96 to 66. An infufion of from half a drachm to three drachms was given for a dofe; it ufually produced flight giddinefs; fometimes naufea, and an inclination to fleep. Dr. Home thus gives us his opinion of this active plant.

"It appears to be one of the moft powerful fedatives which we have, as in moft of the trials it made the pulfe fo remarkably flow, and in one reduced it 38 beats. By fedatives I mean fuch medicines as make the pulfe flower, though not weaker. Such have not been much attended to by phyficians; and, in moft experiments, if the pulfe was not quickened, the experimenter declared they had no effect on the pulfe; never fufpecting that there was a numerous clafs of bodies which rendered it flower, and had as powerful effects on the fyftem as the former. This has been the caufe of many falfe conclufions. I have attended much to this clafs of fedatives, have found it very extenfive, and poffeffing, in common, many effects on the healthful and morbid body. Such medicines appear to be ufeful in a number of difeafes, as they diminifh the fenfibility of the nervous fyftem."*

It is very probable that, among the different fpecies of Digitalis, there may be found fome which fhall act in a much milder manner than the *purpurea*, and yet be equally efficacious. According to the lateft arrangement, thefe fpecies are as follow: viz. 1. Digitalis purpurea; 2. Digitalis minor; 3. Digitalis thapfi; 4. Digitalis lutea; 5. Digitalis ambigua; 6. Digitalis ferruginea; 7. Digitalis obfcura; 8. Digitalis canarienfis; 9. Digitalis fceptrum; 10. Digitalis orientalis; 11. Digitalis cochinchinenfis; 12. Digitalis finenfis.†

When the name and firft defcription of this plant were given by *Fufchius*, only two fpecies were known, the *purpurea* and the *lutea*,‡ and even of thefe the latter is but little attended to at

* Home's Clynical Experiments, third Edition, p. 149.

† Vide Profeffor Martyn's Edition of Miller.

‡ Duum eft generum, una enim purpureos obtinet flores, ideoque Digitalem purpuream appellavimus. Altera luteos habet flores, ob id Digitalis lutea dicta nobis eft.

at prefent, though growing in moft parts of Europe. It was cultivated by Gerarde in 1596. In the Memoirs, however, of the Royal Academy of Berlin, volume for 1794 and 1795, a manufcript differtation by Dr. Careno of Vienna, is announced by M. Mayer, which treats of the efficacy of the *Digitalis lutea* in dropfy. We are told that Dr. Careno has found that this fpecies of the plant poffeffes a ftronger diuretic power than the *Digitalis purpurea, without any of its noxious effects*; and that he has fucceeded in curing dropfical patients with Digitalis lutea, after the moft powerful diuretics had failed.

A poffibility here offers itfelf of difcovering the fedative powers of the Digitalis, detached, in a great meafure, from the noxious principle accompanying the fpecies purpurea.

I have thus, Gentlemen, as far as at prefent lies in my power, attempted, from my own experience, to afcertain the value of this active agent in confumption; and if I may be allowed, in this ftage of the enquiry, to form an inference from the practice, it would be, that in the Digitalis we have difcovered a powerful and generally fuccefsful barrier againft the ravages of this deftructive difeafe; and that, of courfe, it will ultimately prove, if reforted to in due time, one of the greateft bleffings which medicine has conferred upon mankind.

To have contributed fomething toward mitigating the evils of humanity, is to have opened a fource of the pureft individual pleafure; and though in carrying my wifhes into execution, I may be occafionally injured by the fpirit of uncandid controverfy, yet fhall the temporary uneafinefs of wounded feeling weigh little in the fcale; for, in whatever fituation I may be in future placed, whether in ficknefs, in poverty, or in prifon, I will call to mind the exertions I have now made, and be content.

### *To the Editors of the Medical and Phyfical Journal.*

GENTLEMEN,

